# Moxibustion therapy for treating psoriasis vulgaris: A protocol for systematic review and meta-analysis

**DOI:** 10.1097/MD.0000000000025250

**Published:** 2021-03-26

**Authors:** Jiahua Zou, Gang Huang, Chuxiang Hu, Juan Yan, Feiyan Zhang, Haiyong Shi, Xian Yuan, Jiajun Fu, Liping Gong

**Affiliations:** a Jiangxi University of Traditional Chinese Medicine; b Affiliated Hospital of Jiangxi University of Traditional Chinese Medicine, Nanchang, China.

**Keywords:** moxibustion, protocol, psoriasis vulgaris, systematic review

## Abstract

**Background:**

Psoriasis vulgaris (PV) is an immune-mediated skin disease, which has seriously affected the quality of life of patients. At present, moxibustion therapy has been widely used in the treatment of PV. The purpose of this study is to provide high-quality evidence-based medicine to evaluate the effectiveness and safety of moxibustion for PV.

**Methods:**

We will search the following Electronic databases from their inceptions to February 2021 without any language limitation: PubMed, Embase, Cochrane Library, China National Knowledge Infrastructure, WangFang Database, Chinese Science Journal Database, Chinese Biomedical Literature Database. What's more, the grey literature and the references of all included literature will also be retrieved manually. Any clinical randomized controlled trials (RCTs) related to moxibustion therapy for PV will be taken into. In order to complete data synthesis and assess the risk of bias, we will use the RevMan V.5.3 software.

**Results:**

This systematic review will provide an assessment of the current state of moxibustion for PV, aiming to assess the efficacy and safety of moxibustion for patients with PV.

**Conclusion:**

This systematic review will establish convincing evidence to prove the effectiveness and safety of moxibustion for PV.

**INPLASY registration number:**

INPLASY202120008.

## 1 Introduction

Psoriasis is a recurrent chronic inflammatory skin disease, with a global prevalence of about 1% to 3%, affects an estimated 125 million people worldwide.^[1,2]^ Psoriasis vulgaris (PV), accounting for about 85% to 90%, the most common subtype of all manifestations of psoriasis. The main clinical symptoms of PV are erythematous, scaly patches or plaques, sharply demarcated, substantial pruritus.^[3]^ Moreover, while PV can occur anywhere on the body, commonly affected areas include the scalp, trunk, gluteal fold, elbows, and knees.^[4]^ Patients with PV are not only at higher risk for cardiovascular disease and metabolic disease than the general population, but also often associated with other comorbidities such as eye diseases, inflammatory bowel disease, liver disease, kidney damage, depression, cancer.^[5,6]^ According to statistics, in adults, the incidence of PV varied from 30.3 per 100,000 person years in Taiwan to 321.0 per 100,000 person years in Italy.^[7]^ In addition, the prevalence of PV increased from 0.123% in 1984 to 0.47% in 2009 in China.^[8]^ At the same time, the incidence of PV is rising year by year due to changes in lifestyle and diet, which brings greater challenges to clinical treatment, and PV can also put patients bear heavy psychological pressure such as anxiety, depression, and affects their quality of life.^[9,10]^

To date, there is no consensus on the etiology and pathogenesis of PV. Currently, the first-line treatment in the clinic mainly includes topical therapy (eg, topical corticosteroid, vitamin D analogue, calcineurin inhibitor or phototherapy), conventional systemic drug therapy (eg, methotrexate, ciclosporin, acitretin or fumarates) and biological agents (eg, adalimumab, etanercept or infliximab) according to international guidelines.^[11,12]^ Although these treatment methods have been effective in rash relief in the short-term, but it is difficult to obtain satisfactory results, and the cost is high, usually accompanied by various adverse events in the long term.^[13]^ Therefore, there is an urgent need to find a safer, more effective, and low-cost alternative therapy to treat PV.^[14]^

Moxibustion therapies, as one of the traditional Chinese therapies, include governor vessel moxibustion, cotton moxibustion, medicated thread moxibustion, and thermal moxibustion. Based on the traditional Chinese medicine (TCM) theory of blood stasis, for the PV, the moxibustion therapy can warm channels and expel the cold, promoting blood circulation and dredge meridians, and has the characteristics of high safety and low cost. Therefore, it is widely used in the treatment of PV and has become a hot spot in clinical research, with satisfactory results.^[15–17]^ However, as the lack of clinical systematic evaluation and evidence-based medicine evidence. This study uses the method of evidence-based medicine to evaluate the efficacy and safety of moxibustion therapy in the treatment of PV, and provides a basis for further improving the clinical efficacy of PV patients.

## 2 Methods

### 2.1 Study registration

This system review agreement has been registered on the INPLASY website (registrationnumber:INPLASY202120008). (https://inplasy.com/inplasy-2021-2-0008/). And according to the system review and meta-analysis protocol (PRISMA-P) 2015 statement of the preferred report project to guide the completion of this agreement.^[18]^

### 2.2 Inclusion criteria

#### 2.2.1 Type of studies

All randomized controlled trials (RCTs) of moxibustion treatment for PV will be included without any blind method and language limitation. Other types of studies such as non-RCTs, animal experimental research will be excluded.

#### 2.2.2 Type of participants

Participants with physician-diagnosed PV will be included in this review. There will be no restriction on age, sex, ethnic group, the intensity, or duration of symptoms. Those patients combined with other basic diseases will be excluded.

#### 2.2.3 Type of interventions

##### 2.2.3.1 Experimental interventions

Different types of moxibustion therapy will be included (e.g., traditional simple moxibustion, indirect moxibustion, direct moxibustion, governor vessel moxibustion, cotton moxibustion, medicated thread moxibustion). Moxibustion as the main part of the combined therapy will also be included. There will be no limited to the moxibustion materials or frequency of intervention or acupuncture points.

##### 2.2.3.2 Control interventions

The control group could gain guideline-recommended conventional treatment. Other types of interventions such as a placebo or moxibustion will be excluded.

#### 2.2.4 Types of outcome measures

Clinical efficacy and psoriasis area and severity index (PASI) will be accepted as the primary outcomes. The total clinical effective rate is obtained by adding the cure rate and effective rate.

##### 2.2.4.1 Secondary outcomes

The secondary outcomes mainly consist of the following aspects:

1. Itchy (VAS).
2. Symptom score according to the evaluation standard of Chinese medicine.
3. Dermatological quality of life index (DLQI).
4. Hamilton anxiety scale (HAMA).
5. Adverse events.

### 2.3 Exclusion criteria

Exclusion criteria in this review as follows:

1. Select the latest one among the repeated publications.
2. The participants in the study for pregnant and lactating women.
3. Other subtypes of psoriasis such as joint psoriasis, pustular psoriasis, erythroderma psoriasis.
4. Documents whose full text cannot be obtained from various sources.
5. Combined with other serious organic diseases or mental diseases.

### 2.4 Search methods for the identification of studies

#### 2.4.1 Data sources

The following electronic databases will be searched from the inception to February, 2021 without any blind method and language limitation: PubMed, Embase, Cochrane Library, China National Knowledge Infrastructure, WangFang Database, Chinese Science Journal Database, Chinese Biomedical Literature Database What's more, the grey literature, and the references of all included literature will also be retrieved manually.

#### 2.4.2 Search strategy

Search terms consist of disease (psoriasis or plaque psoriasis or psoriasis vulgaris or psoriases or baibi), intervention (moxibustion or indirect moxibustion or direct moxibustion or governor vessel moxibustion or cotton moxibustion or medicated thread moxibustion), and study types (randomized controlled trial or random trials or controlled clinical trial). The search strategy for PubMed is shown in Table 1.

**Table 1:** Search strategy used in PubMed database.

Order	Search terms
#1	psoriasis [Mesh]
#2	Plaque psoriasis [title/absteace]
#3	psoriasis vulgaris [title/absteace]
#4	psoriases [title/absteace]
#5	Baibi [title/absteace]
#6	#1 or #2 or #3 or #4 or #5
#7	Moxibustion
#8	Indirect moxibustion [Mesh]
#9	Direct moxibustion [title/absteace]
#10	Governor vessel moxibustion [title/absteace]
#11	Cotton moxibustion [title/absteace]
#12	Medicated thread moxibustion [title/absteace]
#13	#7 or #8 or #9 or #10 or #11 or #12
#14	Randomized controlled trials [title/absteace]
#15	Controlled clinical trial [title/absteace]
#16	Random trial [title/absteace]
#17	#14 or #15 or #16
#18	#6 and #13 and #17

### 2.5 Data collection

#### 2.5.1 Studies selection

**Figure 1:**
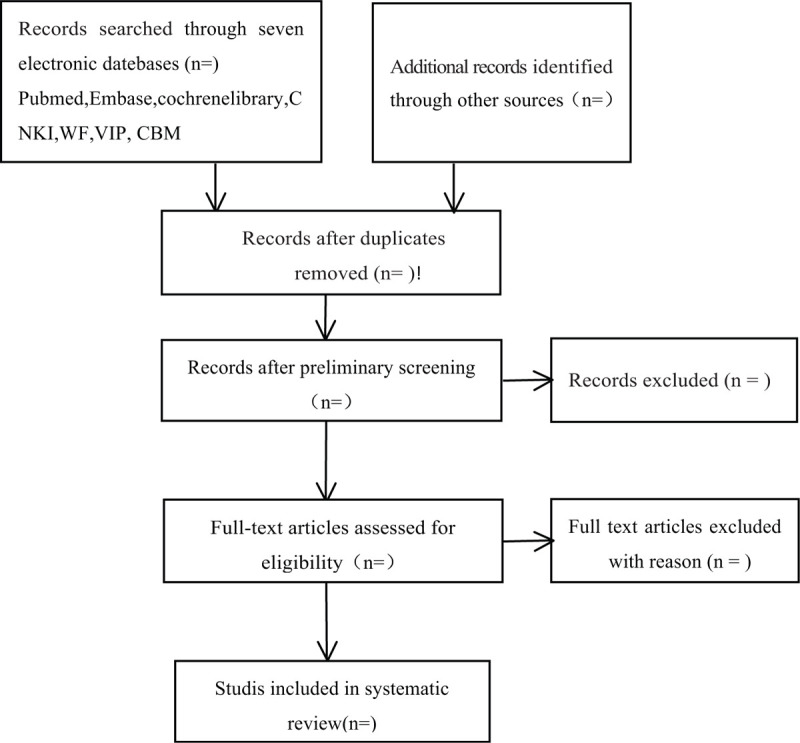
Flow diagram of study selection process.

After completing the search, the researchers will import the search results into the noteexpress 3.2.0 software and eliminate the duplicate literature. First of all, 2 researchers (JY and XY) independently browse the title and abstract of the literature to exclude the literature that did not meet the inclusion criteria. Then, we will further read the full text of the document to obtain the higher-quality literature. After that, 2 researchers will cross-check the selection results. If there are disagreements, we will contact the first author of the document or invite the third reviewer (GH) to resolve it, and finally determine the documents that need to be included. The literature selection process is shown in Fig. 1.

#### 2.5.2 Data extraction and management

After confirm the final document, the data of the document will be extracted independently by the 2 reviewers (HYS and JJF), then further cross-check. Any disagreement will be resolved by discussions between the 2 reviewers, if still cannot be resolved, the final judgment will be decided by the third reviewers (CXH). The extracted data mainly includes the following information: basic information (e.g., first author, published time, Literature sources, study location), study design (e.g., study type, participant characteristics, sample size, interventions, duration of intervention, outcomes, adverse events, and other relevant characteristics in the full text).

### 2.6 Data analysis

#### 2.6.1 Assessment of risk of bias in included studies

To evaluate the bias of the included literature, we will use the Cochrane risk assessment tool. The Cochrane evaluation entries mainly consist of the following aspects: random sequence generation, allocation concealment, blinding of participants and researchers and outcome assessors, incomplete outcome data, selective outcome reporting, and other bias. For each item, risk of bias in divides it into 3 levels: low risk, high risk, and unclear risk. If there is any disagreement after the assessment, we will deal with it through rechecking the source papers or discussion with the third investigator (GH).

#### 2.6.2 Quantitative data synthesis

RevMan software (Version5.3, Copenhagen: The Nordic Cochrane Centre, The Cochrane Collaboration, 2014) will be applied in this meta-analysis and quantitative data synthesis. If the analysis data belong to continuous data, we will use mean difference (MD) or standard mean difference (SMD) with 95% confidence interval (95% CI) as the effect measure. Meanwhile, the dichotomous data will be calculated with the odds ratio (OR) with 95% CI.

#### 2.6.3 Dealing with missing data

To obtain complete information, missing data will be obtained by contacting the author of the study by phone or email. If the author cannot be contacted, we will only analyze the existing data and further discuss the potential impact of missing data.

#### 2.6.4 Assessment of heterogeneity

We will use the Q test and *I*^2^ test in RevMan 5.3 software to perform heterogeneity test and quantitative analysis for each result index. According to the heterogeneity test results of the study, fixed or random effects models are used respectively. If there is no heterogeneity among the statistical results (*P* > .10, *I*^2^ < 50%), the fixed-effect model will be used; otherwise, the random effects model will be applied in this meta-analysis.

#### 2.6.5 Subgroup analysis

If an obviously heterogeneity is detected between studies, subgroup analyses will be performed according to the different types of moxibustion therapy and treatment time.

#### 2.6.6 Sensitivity analysis

In order to acquire a stable result, we will conduct sensitivity analysis if there is high heterogeneity between studies.

#### 2.6.7 Assessment of publication bias

The main function of the funnel plot is to assess whether there is publication bias in our studies. We will use it to estimate publication bias if the number of included studies is sufficient (>10 studies).

#### 2.6.8 Summary of evidence

Under the guidance of the Grading of Recommendations Assessment, Development, and Evaluation System (GRADE), we will evaluate the quality of evidence for each main outcome. Each study will be evaluated by 2 reviewers from the following 4 aspects: high quality, medium quality, low quality, and very low quality.^[19]^

#### 2.6.9 Ethics and dissemination

Since this systematic review not only does not involve the personal privacy of participants, it will also be published in peer-reviewed publications. Therefore, Ethics approval is not required.

## 3 Discussion

Psoriasis is a skin disease closely related to the season, with peaks in the late winter/early spring and troughs in the late summer/early autumn.^[20]^ The pathogenesis of the disease is very complicated with many pathogenic factors, it is mostly considered to be related to infection, genetics, immunity, diet, and environmental factors.^[21,22]^ At present, western medicine, as a first-line treatment, is used to treat local or systemic diseases, which can relieve clinical symptoms, but has a high recurrence rate. Apart from the high treatment costs, the adverse events caused by these treatment methods are very harmful, and affects the quality of life of patients seriously.^[13]^ However, moxibustion therapy has the characteristics of high safety, less complications, and low cost. It uses TCM acupuncture theory as the guiding ideology, with warm channels and expel the cold, promoting blood circulation, and dredge meridians. Previous studies have proved that moxibustion have good application prospects for the treatment of PV, but there is a lack of scientific and systematic evidence to evaluate its efficacy. Therefore, it is worthy of systematically evaluating them to establish convincing evidence to prove the effectiveness and safety of moxibustion therapy for PV. However, this systematic review may also has some limitations and insufficiency. The number of RCTs included in this study may be small, which will affect the reliability of the results of the study. What's more, in the process of moxibustion treatment, there may be significant differences in the selection of acupoints and the frequency of intervention.

## Author contributions

**Conceptualization:** Jiahua Zou, Gang Huang.

**Data curation:** Juan Yan, Xian Yuan.

**Formal analysis:** Chuxiang Hu.

**Investigation:** liping Gong.

**Methodology:** Jiahua Zou.

**Project administration:** Feiyan Zhang.

**Software:** Haiyong Shi, Jiajun Fu.

**Writing – original draft:** Jiahua Zou, Feiyan Zhang.

**Writing – review & editing:** Gang Huang, Chuxiang Hu, liping Gong.
